# Integrating Factor Analysis and a Transgenic Mouse Model to Reveal a Peripheral Blood Predictor of Breast Tumors

**DOI:** 10.1186/1755-8794-4-61

**Published:** 2011-07-22

**Authors:** Heather G LaBreche, Joseph R Nevins, Erich Huang

**Affiliations:** 1 Institute for Genome Sciences and Policy, Duke University, 101 Science Drive, Durham, 27710, NC USA; 2 Department of Molecular Genetics and Microbiology, Duke University, 268 CARL Building, Research Drive, Durham, 27710, NC USA; 3 Department of Surgery, Duke University Medical Center, 2301 Erwin Road, Durham, 27705, NC USA; 4 Sage Bionetworks, 1100 Fairview Avenue North, Seattle, 98109, WA USA

## Abstract

**Background:**

Transgenic mouse tumor models have the advantage of facilitating controlled *in vivo *oncogenic perturbations in a common genetic background. This provides an idealized context for generating transcriptome-based diagnostic models while minimizing the inherent noisiness of high-throughput technologies. However, the question remains whether models developed in such a setting are suitable prototypes for useful human diagnostics. We show that latent factor modeling of the peripheral blood transcriptome in a mouse model of breast cancer provides the basis for using computational methods to link a mouse model to a prototype human diagnostic based on a common underlying biological response to the presence of a tumor.

**Methods:**

We used gene expression data from mouse peripheral blood cell (PBC) samples to identify significantly differentially expressed genes using supervised classification and sparse ANOVA. We employed these transcriptome data as the starting point for developing a breast tumor predictor from human peripheral blood mononuclear cells (PBMCs) by using a factor modeling approach.

**Results:**

The predictor distinguished breast cancer patients from healthy individuals in a cohort of patients independent from that used to build the factors and train the model with 89% sensitivity, 100% specificity and an area under the curve (AUC) of 0.97 using Youden's J-statistic to objectively select the model's classification threshold. Both permutation testing of the model and evaluating the model strategy by swapping the training and validation sets highlight its stability.

**Conclusions:**

We describe a human breast tumor predictor based on the gene expression of mouse PBCs. This strategy overcomes many of the limitations of earlier studies by using the model system to reduce noise and identify transcripts associated with the presence of a breast tumor over other potentially confounding factors. Our results serve as a proof-of-concept for using an animal model to develop a blood-based diagnostic, and it establishes an experimental framework for identifying predictors of solid tumors, not only in the context of breast cancer, but also in other types of cancer.

## Background

The concept of using peripheral blood cells (PBCs) as the source of information to predict the presence of cancer relies on the natural role of these cells in responding to physiologic and pathologic changes coupled with the potential of genome-scale transcriptome data to identify patterns of expression that reflect a given phenotype. Using this approach, researchers have described gene expression signatures that distinguish cancer patients from healthy individuals in a number of different types of cancer [1-7]. Twine et al. [6] generated a peripheral blood gene expression signature that could distinguish renal cell carcinoma patients from healthy volunteers. Likewise, Osman et al. [3] reported a whole-blood gene expression signature that could distinguish bladder cancer patients from controls. Similar studies have also been conducted in the contexts of colorectal cancer [2], melanoma [7], non-small cell lung cancer [5] and even breast cancer [1,4]. A few of these studies have also suggested that blood transcriptome profiling could be used to not only distinguish cancer patients from normal, healthy volunteers, but also differentiate among tumor types [3,6]. Taken together, these studies highlight the potential of peripheral blood signatures as cancer biomarkers.

Our previous work has made use of expression data to develop predictors of tumor outcome, activation of various cell signaling pathways, and response to targeted therapies. In each instance, the ability to accurately define the extremes of particular phenotypes (short versus long survival, pathway off versus pathway on) has been useful in the development of informative expression signatures. These extreme cases represent an opportunity to generate gene expression data, which can be used to train a prediction model. This prediction model can then be applied to a wide spectrum of phenotypes [8-10]. Using a similar strategy in the context of signatures from peripheral blood cells, we hypothesized that a mouse model of breast cancer might offer the best opportunity to maximize the distinction between animals with and without a tumor. As such, we have made use of a transgenic mouse model of breast cancer, which gives rise to spontaneous mammary tumors with approximately 100% penetrance in multiparous mice. Our approach was to develop latent factor models in the mouse PBC transcriptome that distinguish controls from mice with advanced mammary tumors, identify orthologous transcripts on human microarrays and use this information as the starting point for a predictive model of human breast tumors.

This strategy overcomes many of the limitations of earlier studies by using the model system to reduce noise and identify transcripts associated with the presence of a mammary tumor over other potentially confounding factors. Our results serve as a proof-of-concept in using an animal model to develop a blood-based diagnostic and establish an experimental framework for identifying possible breast cancer signatures (factor models).

Breast cancer is the most common cancer for women across the world. According to Kamanger et al. [11], breast cancer is responsible for an estimated 1,300,000 new cases and 465,000 deaths each year. The most common breast cancer screening modality is mammography, which aims to identify breast tumors before they become symptomatic. Breast tumors identified during mammographic screening tend to be smaller and are less likely to have spread to distant sites within the body. This has important implications for a patient's prognosis. Relative survival rates for women in the United States diagnosed with localized cancer are over 98%, but for women whose cancer has metastasized, it is as low as 23% [12], emphasizing the importance of detecting tumors at an early stage so that treatment can be initiated. Despite the success of mammography in reducing breast cancer mortality, there are several scenarios that illustrate the need for new and innovative methods of breast cancer detection. The growth rate of breast tumors varies significantly from patient to patient [13], meaning that even a biannual mammography screening program will miss some fast-growing cancers [14,15]. Additionally, mammographic sensitivity is lower in young women and women with dense breasts [16-18]. Sensitivity is also lower in women who are at higher risk of developing breast cancer because of a genetic predisposition or family history [19,20].

## Methods

### Mouse housing and maintenance

Animal use and husbandry was in accordance with institutional and federal guidelines. Eran Andrechek generated the transgenic mice [21] through standard methods based on the model described by Leder et al. [22]. Female MMTV/*c-MYC *transgenic mice expressed the *c-MYC *proto-oncogene or a more stable point mutation variant (T58A) of the gene under the control of the hormone-responsive MMTV long terminal repeat (LTR) in an FVB/NJ background (Jackson Laboratories, Bar Harbor, ME). The hormones released during pregnancy and lactation have been shown to enhance expression of the oncogene. Thus, the mice were maintained in a continuous breeding program. Mice were monitored twice weekly for tumor development by palpation and tumors were measured twice weekly. Once the tumors reached 3 cm^3 ^the animals were sacrificed and tissue was obtained to confirm the tumors by histological analysis. As a control, female mice of the same age and background strain were maintained in the same facility and under the same breeding conditions as their transgenic counterparts.

### Mouse blood collection, leukocyte isolation and RNA extraction

Blood (50-250 μL, based on weight of the mouse) was collected from MMTV/*c-MYC *female mice and controls at regular intervals (approximately once per month) and again prior to euthanization (average age 431 days). Samples were collected using the submandibular (cheekpouch) method. Briefly, the vein that drains the face and cheek area was punctured by a lancet (GoldenRod animal lancet, MEDIpoint, Inc., Mineola, NY) and blood was captured in BD Microtainer™ tubes with potassium-EDTA anticoagulant (Becton Dickinson, Franklin Lakes, NJ). Tubes were immediately inverted 10-12 times and placed on ice. Following erythrocyte lysis, total RNA was isolated from the leukocyte pellet using the QIAamp RNA Blood Mini Kit (Qiagen, Valencia, CA). Alternatively, the leukocyte pellet was immediately lysed, homogenized and stored at -80°C. RNA was isolated at a later date using the protocol described above or the adapted protocol associated with the QIAamp RNA Blood Mini Kit for use in the QIAcube (Qiagen, Valencia, CA). The quantity of RNA was assessed by absorbance at 260 nm by the NanoDrop ND-1000 (Thermo Fisher Scientific, Wilmington, DE). RNA quality was assessed by Agilent 2100 Bioanalyzer RNA 6000 PicoChip (Agilent, Palo Alto, CA). Purified total RNA (200 ng) was amplified using NuGEN Ovation™ RNA Amplification System (NuGEN Technologies, Inc., San Carlos, CA). Amplified RNA was labeled and hybridized onto Affymetrix M420 2.0 GeneChip oligonucleotide arrays (Affymetrix, Santa Clara, CA) according to the manufacturer's instructions.

### Complete blood count and differential

Whole blood was collected from mice immediately following euthanization. The samples were collected from the hepatic portal vein using a 3 mL syringe and a 19-gauge needle. The sample was immediately transferred to a collection tube containing K_2_EDTA, inverted 10-12 times and placed on ice. Samples (130 μL minimum) were delivered to the Duke University Medical Center Veterinary Diagnostic Laboratory and were analyzed within 8 hours of collection using a Cell-Dyn 3700 Hematology Analyzer. Manual differentials were performed as necessary.

### Quantitative RT-PCR

RNA was isolated from mouse PBCs as previously described. We analyzed PBC RNA from each of the 5 transgenic lines and wildtype FVB/N females in triplicate. We also analyzed mammary tumor RNA from a transgenic mouse and mammary gland RNA from a wildtype lactating female. We used the following primer pair to amplify the *c-MYC *transgene: 5'-CTGTCCATTCAAGCAGACGA-3' and 5'-GTATGGGTACCCTGCACCAG-3'. Total *MYC *(transgenic and endogenous) was amplified using the following primer pair: 5'-GCCATAATTTAACTGCCTCAAA-3' and 5'-CCTATTTACATGGGAAAATTGGA-3'. All reported values represent the threshold change as compared to the housekeeping gene beta-actin and are relative to *MYC *expression in the wildtype lactating mammary gland.

### Comparison of MYC transcript levels from microarray analysis

We identified probes from the M430 2.0 Affymetrix GeneChip related to the myelocytomatosis oncogene (avian v-myc myelocytomatosis viral oncogene homolog) by using the NetAffx Query [23] tool. We then compared the average signal intensity of these probes across all 4 groups of mice based on MAS5 normalized data: 1) FVB virgin controls; 2) FVB age-matched controls; 3) MMTV/*c-MYC *pre-tumor and 4) MMTV/*c-MYC *post-tumor.

### Patient samples

Peripheral blood mononuclear cell (PBMC) samples were collected from women with a suspect initial mammogram prior to undergoing a diagnostic biopsy procedure to determine whether the detected abnormality was benign or malignant. In total, we collected blood from 57 women with a diagnosis of breast cancer and 37 with a benign diagnosis. We also collected blood from 31 women with normal initial mammograms as negative controls and 15 breast cancer patients following surgery. All breast cancer patient samples were collected at the Duke University Medical Center under an institutional review board-approved protocol (Duke eIRB#12025) after obtaining informed consent and were provided by Dr. Jeffrey Marks. PBMC samples from patients with various gastrointestinal cancers (n = 15) were collected and stored at Duke University Medical Center under institutional review board-approved protocols (Duke eIRB#12010 and 12025) and were provided by Dr. Jeffrey Marks. Peripheral blood leukocyte samples from patients with brain tumors (n = 7) were provided by Dr. John Sampson and were collected by leukapheresis under Duke eIRB#00003877 and #00009403.

### Human blood collection, PBMC isolation and RNA extraction

Blood samples (8 mL) were drawn into BD Vacutainer™ CPT™ Cell Preparation tubes with sodium citrate (Becton Dickinson, Franklin Lakes, NJ) by routine venipuncture, inverted 8-10 times to mix, labeled with a patient identification number and transported to the laboratory at room temperature for processing within 2 hours. Tubes were mixed again immediately prior to processing. Tubes were centrifuged for 30 minutes at 2,500 rpms in a centrifuge with a swinging bucket rotor. The plasma layer was removed, and then the remaining buffy coat was poured into a 15 mL conical tube. Next, 5 mL of chilled phosphate buffered saline (PBS) with 2% fetal bovine serum (FBS) was added to the CPT tube, which was capped and inverted to mix. The contents were poured into the same 15 mL conical tube, which was then centrifuged for 10 minutes at 1200 rpms at room temperature. The supernatant was then aspirated and the pellet was resuspended in 1 mL of freezing media (RPMI with 20% DMSO and 20% FBS). The cells were then transferred to a cryovial (Nunc, Roskilde, Denmark) and placed in a freezer box at -80°C. After 24 hours samples were transferred to liquid nitrogen storage. Total RNA from peripheral blood mononuclear cells was isolated using the Ambion Ribopure™-Blood kit (Ambion, Austin, TX). The quantity of RNA was assessed by absorbance at 260 nm by the NanoDrop ND-1000 (Thermo Fisher Scientific, Wilmington, DE). RNA quality was assessed by Agilent 2100 Bioanalyzer RNA 6000 NanoChip (Agilent, Palo Alto, CA). Purified total RNA (2.5 μg) was labeled and hybridized onto Affymetrix U133 Plus 2.0 GeneChip oligonucleotide arrays (Affymetrix, Santa Clara, CA) according to the manufacturer's IVT 1-cycle processing protocol.

### Generation of a mouse mammary tumor predictor

The entire mouse gene expression dataset was standardized employing principal components of the Affymetrix control probe sets [24]. Then, we randomly divided the samples into roughly equal-sized training (n = 46) and validation cohorts (n = 47). Next, we then performed a sparse ANOVA on the training data set (45,101 Affymetrix probes) to identify 4,276 genes that were significantly differentially expressed between the control and tumor-bearing mice (based on a posterior probability of ≥0.99). We used Bayesian factor regression modeling (BFRM) [25] to identify 49 factors, or linear combinations of genes, in our training dataset. BFRM allowed us to discover underlying structure and make class predictions within a high-dimensional data set by using sparse statistical models. To model dependencies among many genes, sparse factor loading matrices are used to generate latent factor models (in other words, we make the sparsity assumption that few genes among the tens of thousands assayed will reflect the underlying biology). Operationally, this is accomplished by randomly seeding sparse regressions that define candidate modules with genes. Modules must meet a posterior probability threshold for inclusion, while candidate genes must also meet a posterior probability threshold for inclusion in a module. We utilized the shotgun stochastic search (SSS) [26] method to identify small subsets of the factors that predicted tumor status within the training set, which included samples from 14 control mice and 32 transgenic tumor-bearing mice. SSS is a neighborhood-based procedure that searches a model space by (1) using the current model to define a neighborhood of proposed models, (2) evaluating each proposal model within this neighborhood in parallel, (3) choosing a new model from these choices. It is useful in situations where there is uncertainty about predictors in the model by penalizing model dimension. Using our training set, we generated 5000 possible predictive models and used model averaging (based on median posterior marginal probability) of the top 200 predictive models. Model averaging has been shown to perform better than algorithms that use the single best model for prediction because it gives a truer estimation of uncertainty [27]. Then the resulting fitted regression models were used to predict tumor status in a separate validation set of mouse PBC samples from 14 control mice and 33 transgenic tumor-bearing mice. For fully versioned and annotated source code and data objects, please see the QUADRA repositories "mouse-data-prep" and "mouse-classification-model" at [28]. The data discussed in this publication have been deposited in NCBI's Gene Expression Omnibus and are accessible through GEO Series accession number GSE27567 [29].

### Development of a human breast tumor predictor

**Figure 1:**
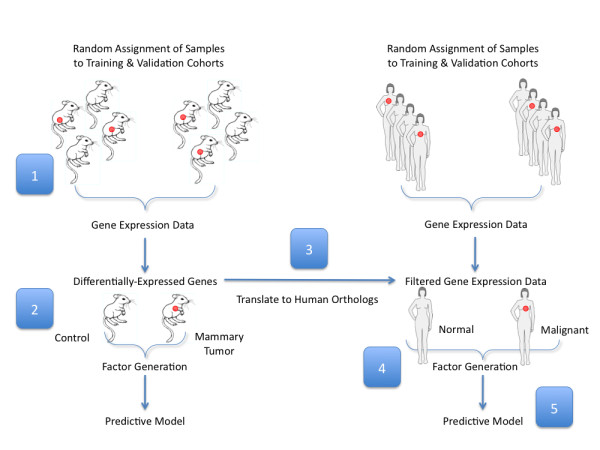
**Experimental Design**. To generate a human breast tumor predictor we took the following steps: (1) randomly divided samples into training and validation cohorts, then analyzed gene expression in PBC samples collected from tumor-bearing transgenic mice versus tumor-free controls that are matched for age and parity; (2) applied a sparse ANOVA to the training set to identify 4,276 mouse Affymetrix probes with at least 0.99 posterior probability (these probes were used to independently generate a set of 49 factors that were used to develop a predictive model based on the mouse gene expression data); (3) translated these into 2,595 orthologous human probes; (4) applied BFRM to this subset of 2,595 probes to yield 26 factors from a training set of 30 human PBMC samples; (5) used SSS to build 5000 possible predictive models from the training set, then projected these into a separate validation set of samples.

In order to generate a human breast tumor predictor, we utilized the list of significantly differentially expressed genes identified previously from the mouse gene expression data. This list of 4,276 Affymetrix Mouse Genome 430 2.0 probe identifiers was translated into 2,595 orthologous human probes (Affymetrix HU133 Plus 2) and used to filter our human dataset, which was normalized employing principal components of the Affymetrix control probe sets. We divided our samples into completely independent training and validation sets. The training set consisted of 30 PBMC samples (10 breast cancer patients, 10 patients with benign breast abnormalities and 10 healthy individuals). To minimize potential batch effects, these samples were hybridized in 4 batches that each included samples from all three phenotypic categories: healthy individuals, patients with benign breast abnormalities and patients with breast tumor. We used BFRM to identify 26 factors, or linear combinations of genes, in the training dataset. The 26 factors represent common patterns of expression within the set of 2,595 genes. We utilized the SSS method to identify small subsets of the factors that were predictive of breast tumor status within the training set. Using our training set, we generated 5000 possible predictive models and used model averaging (based on median posterior marginal probability) of the top 200 predictive models. Then the resulting fitted regression models were used to predict breast tumor status in a separate validation set of PBMC samples. A schematic of our experimental design can be found in Figure 1.

To evaluate the reproducibility of the predictive model, we performed permutation testing and a swap experiment. The permutation test used the same factors generated from our human PBMC training set. However, we randomly permuted (x100) which samples were assigned to the training and validation sets. The "swap experiment" is based on the recommendations of the MicroArray Quality Control (MAQC)-II study [30] and consisted of swapping the training and validation sets, generating new factors from the original validation set and then validating these new factors in the original training set. Next, we assessed the biological relevance of the factors to the phenotype in question (presence of a breast tumor) by performing two negative controls. First, we used the original factors, but scrambled the phenotypic labels (Normal and Malignant) iteratively, evaluated the predictive models and looked at the distributions of the test characteristics. We performed 200 iterations. Second, we generated a set of mock factors from a publicly available insulin/muscle-biopsy dataset (GSE7146) and projected them into the original training and validation sets. We then compared this predictive model to the human breast tumor predictor generated from the mouse ortholog data. As a validation of our overall approach, we also tested the utility of generating a breast tumor predictor from the human PBMC gene expression data using the same parameters applied to the mouse gene expression data. For fully versioned and annotated source code and data objects, please see the corresponding QUADRA repositories [28]. The data discussed in this publication have been deposited in NCBI's Gene Expression Omnibus and are accessible through GEO Series accession number GSE27567 [29].

### Functional Annotation

To explore the underlying biology of a breast tumor predictor based on peripheral blood gene expression, we analyzed the genes included in the predictor using publicly available annotation tools. To simplify our analysis, we included all 115 unique Affymetrix probes (3 probes were represented in 2 factors each) that comprise the 3 top-performing factors in the predictive model (factors 3, 12 and 14). We used the Affymetrix NetAffx Batch Query tool to translate the 115 HU133 Plus 2.0 Array probe identifiers into 115 gene symbols and descriptions. We used GATHER [31,32] to input the lists of 83 upregulated and 32 downregulated probe identifiers and generate annotations for gene ontology categories, KEGG pathways, protein binding partners and transcription factor binding sites.

### Gene Set Enrichment Analysis

Gene Set Enrichment Analysis (GSEA, version 2.0.5) was used as previously described [33,34]. Briefly, we used the gene set annotation feature of GSEA [35] to identify those gene sets that overlap with the upregulated and downregulated genes in the breast tumor predictor. These gene sets offer insights into the functional annotation of our experimentally derived factors and have the potential to reveal the underlying biology of each factor. We used the list of Affymetrix probe identifiers as the input into the browser's query field. After selecting the appropriate identifier platform (Affymetrix HU133 Plus 2.0), we chose to compute the top 10 overlaps with the each of the following categories of gene sets: C1, which represents 326 positional gene sets; C2, which is a collection of 1,892 curated gene sets; C3, which contains 836 gene sets with a shared, putative cis-regulatory element that is conserved across species; C4, which contains 881 computational gene sets that are defined by mining large collections of cancer-oriented microarray data and C5, which contains 1,454 gene sets annotated by a common GO term. In our efforts to annotate the biological function of each factor, we focused on those gene sets that incorporated a large proportion of the genes within the predictor and had the most statistically significant overlap.

### QUADRA

QUADRA encompasses both an open source distributed version control system 'Git' (Version 1.7.2.2 [36]), an adaptation of the open source Ruby on Rails web hosting platform 'Gitorious' [37] as well as 'good operating principles' in genomic computation. The basic principles of QUADRA encompass the following: (1) all data manipulations start with raw data (as obtained from a core facility or GEO), and all manipulations/interactions with the data are performed and versioned within a scientific computing environment, in this case MATLAB (Version 7.9.0.529, R2009b, Mathworks, Natick, MA), (2) the entire analytic flow is recorded and versioned in source code; therefore, all aspects of factor and model generation are performed programmatically and can be read as MATLAB source code, (3) once an analysis is finalized, its "publish" run is recorded as an HTML file so that the scientific community can reconcile the actual analysis with what is reported and the source code; and (4) all source code and data are available to the public [28].

## Results

### Development of a peripheral blood predictor of mouse mammary tumors

**Figure 2:**
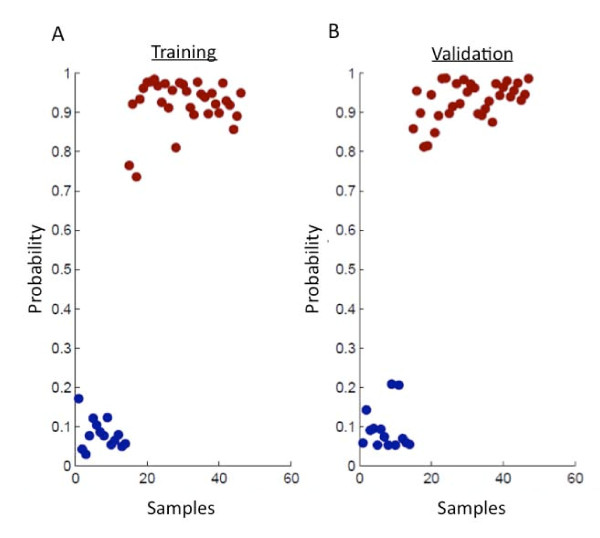
**Generation and validation of the mouse mammary tumor signature**. We generated a mouse mammary tumor predictor based on gene expression of PBCs from a training set of tumor-bearing transgenic mice (n = 32) and nontransgenic tumor-free mice (n = 14). (A) This signature is capable of distinguishing the two classes accurately within the traning data set as shown in the model fit diagram. Blue = healthy tumor-free control mice; red = MMTV/*c-MYC *tumor-bearing mice. (B) Furthermore, this signature was applied to an independent set of PBC samples from transgenic tumor mice (n = 33) and nontransgenic controls (n = 14) to predict the tumor status of each sample. It demonstrated 100% sensitivity and 100% specificity in predicting tumor status, using the optimal threshold of 0.8118 as calculated using Youden's J-statistic.

The overall modeling strategy we employed involves using the mouse model to first identify those genes that are most relevant to the PBC response to a mammary tumor and then using these genes as the basis for developing a human breast tumor predictor. This strategy is outlined in Figure 1. This approach was based on our initial findings that these transcripts were sufficient to develop a robust and accurate predictor of mouse mammary tumors. To generate a predictive model based on peripheral blood gene expression in the mouse, we first randomly divided samples into roughly equal training and validation cohorts. We strictly adhered to maintaining the independence of the training and validation cohorts by performing all model-building steps in the training set. Following this principle, we next used a sparse ANOVA method to identify 4,276 differentially expressed transcripts between transgenic tumor-bearing mice and age-matched controls in the training cohort and subsequently used (BFRM) to generate a collection of 49 factors in the these samples [25]. These factors were used as variables in SSS [26], to generate a predictive model, which was based solely on the training data and then validated in our independent validation cohort. The predictive model performed with 100% sensitivity and specificity in the training set. More importantly, it performed equally well in the validation set, distinguishing samples based on tumor status with 100% sensitivity and specificity (Figure 2).

### Probing the biological basis of a mouse mammary tumor predictor

For these studies, we used the MMTV/*c-MYC *transgenic mouse model. Although the MMTV (mouse mammary tumor virus) promoter is known to control *c-MYC *expression in a mammary-specific manner, we examined *c-MYC *expression in the blood cells of transgenic mice in order to confirm that the peripheral blood signature we observed was not the result of aberrant *c-MYC *expression. We used quantitative RT-PCR to confirm that the transgenic mice used in these experiments have little to no expression of the *c-MYC *mRNA transcript in the peripheral blood cells relative to the high levels observed in the mammary tumors from the transgenic mice. In a second approach, we compared the levels of *MYC *expression among the different groups of mice based on the signal intensity of the Affymetrix probe sets. Our results (Additional File 1) suggest that the peripheral blood signature we observe is not a result of *c-MYC *expression in the peripheral blood cells. In addition, we show that the transgenic and wildtype mice used as the basis for the breast tumor predictor have similar leukocyte profiles, indicating that the observed gene expression changes are not due to a difference in the proportions of the various leukocyte subpopulations (Additional File 2).

### Human Patient characteristics

The training set contained samples from women diagnosed with invasive breast cancer or benign breast abnormalities, as well as samples from women with no evidence of breast cancer. The entire training set (including Benign, Normal and Malignant samples) was used to generate factors. The predictive model was built based on a comparison of the Normal and Malignant samples. The validation set contained samples from women with normal, benign or malignant diagnoses, as well as samples from patients with a variety of gastrointestinal cancers or brain cancer. The clinical and demographic characteristics of the training and validation patient cohorts are broken down into phenotypic subgroups (Normal and Malignant) and reported in Table 1. These two cohorts did not show any statistically significant differences in terms of age, race, gender, diagnosis, BI-RADS score, hormone receptor status or lymph node status. The training cohort consisted of 10 samples from healthy, control individuals (average age of 56 years; range 44-75) and 10 samples from breast cancer patients (average age of 61 years; range 43-85); the validation cohort consisted of 21 samples from healthy, control individuals (average age of 58 years; range 46-80) and 47 samples from breast cancer patients (average age of 59 years; range 32-85). A comparison of patient characteristics based on phenotypic group can be found in Additional File 3. Notably, we ensured that the patient samples in the training cohort were all processed together to minimize technical batch effects.

**Table 1:** Patient characteristics by training and validation data set.

Normal (n = 31)				
**Category**		**Training (n = 10)**	**Validation (n = 21)**	**p-value**

Age (y)				0.5400*
	Mean	55.80	57.81	
	Median	55.00	55.00	
	Max	75.00	80.00	
	Min	44.00	46.00	
Gender				N/A
	Female	20	68	
	Male	0	0	
Race				0.2216†
	White	9	14	
	Other	1	7	
BI-RADS Score				0.2540‡
	0	0	2	
	1	9	13	
	2	1	6	
	3	0	0	
	4	0	0	
	5	0	0	
	6	0	0	

	Incomplete/Unavailable	0	0	

Malignant (n = 57)				

Category		Training (n = 10)	Validation (n = 47)	p-value

Age (y)				0.6444*
	Mean	61.4	58.68	
	Median	56	61	
	Max	85	85	
	Min	43	32	
Gender				N/A
	Female	10	47	
	Male	0	0	
Race				1.0000†
	White	9	14	
	Other	1	7	
BI-RADS Score				0.8695‡
	0	0	2	
	1	0	2	
	2	1	2	
	3	0	0	
	4	3	16	
	5	4	12	
	6	0	2	
	Incomplete/Unavailable	2	11	
HER2				0.2253‡
	Positive	3	5	
	Negative	4	18	
	Unknown	3	24	
ER				0.1036‡
	0 - 2	3	15	
	3 - 8	4	6	
	Unknown	3	26	
PR				0.2844‡
	0 - 2	4	12	
	3 - 8	3	8	
	Unknown	3	27	
Positive Lymph Nodes				0.0685‡
	No LN Sampling	1	23	
	0	5	18	
	1	1	4	
	2	1	1	
	3	0	1	
	9	1	0	

* Mann-Whitney test

† Fisher's exact test

‡ Chi-square test

N/A: Not Applicable

### Development of a peripheral blood predictor of human breast tumors

Based on the performance of the mouse mammary tumor predictor, we sought to use these studies as the foundation for developing a predictor of human breast tumors. To develop this predictor, we started with the list of differentially expressed genes derived from the mouse model and translated them into their human gene orthologs. We used this gene list to filter our human gene expression data. Next, we used the same modeling pipeline employed for the mouse model to generate sparse factors and train a predictive model using shotgun stochastic search. In building the models, we again randomly divided the dataset into independent training and validation cohorts, as previously described, and ensured that factors and the predictive model built from them were generated from the training set alone and completely insulated from the validation set.

**Figure 3:**
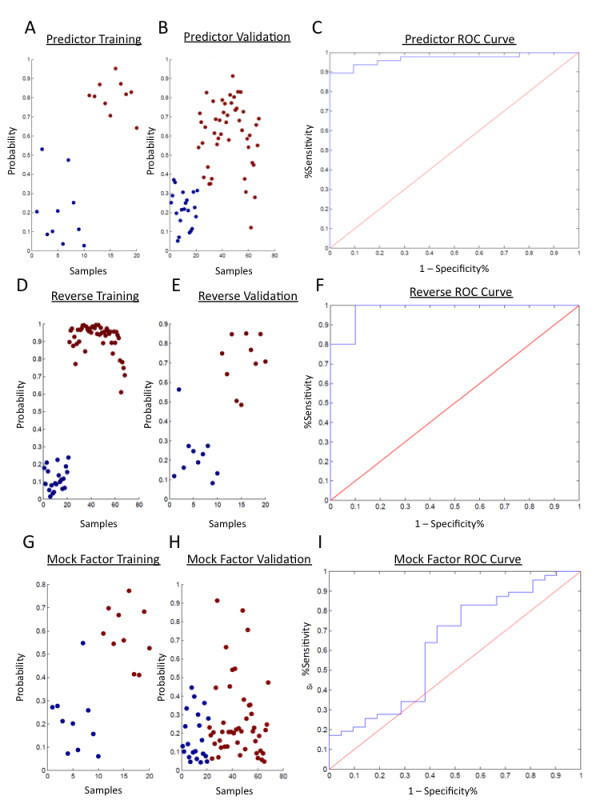
**Gene expression signature predicts human breast cancer**. We generated a human breast cancer predictor from human PBMC samples by using a mouse model of breast cancer to first identify the most informative probes to use in a subsequent factor modeling approach. (A) We generated the predictive model from a training set of healthy cancer-free individuals (n = 10) and patients with invasive breast cancer (n = 10). Blue = normal; red = malignant. An assessment of the model fit shows that this predictor has a robust capacity to discriminate among samples based on breast tumor status with 100% sensitivity and specificity. (B) We then used this predictive model to evaluate an independent set of samples (n = 162) for the capacity to distinguish controls from patients with a diagnosis of malignant breast cancer. This represents an external validation using samples not used in either the factor generation or the model building process. (C) We were able to predict breast cancer status with a sensitivity of 89% and specificity of 100% (AUC = 0.97) as shown in the ROC curve. The optimal threshold was calculated as 0.3760 based on Youden's J-statistic. (D) We then tested the validity of our modeling strategy by swapping the training and validation sets. New factors were generated based on the original validation set and new models were generated. The model fit diagram shows the ability to generate a robust model from the original validation set. (E) This new model was validated in the original training set. (F) It demonstrated a sensitivity of 100% and a specificity of 90% (AUC = 0.98). As a negative control, we generated mock factors from a publicly available dataset that was biologically unrelated to breast tumor status. We then projected these factors into the training (G) and validation (H) sets. (I) The sensitivity was 83% and specificity was 48% (AUC = 0.63).

The performance of the derived predictive model in distinguishing controls from cancer patients is shown in Figure 3 (A-C). The predictor showed robust capacity to distinguish between the two groups in the training set. Moreover, we were able to predict tumor status in the validation set with 89% sensitivity and 100% specificity (as calculated using Youden's J-statistic to obtain a threshold of 0.3760). The diagnostic characteristics of this predictive model were tested by a receiver-operator characteristic (ROC) curve, which demonstrated an area under the curve (AUC) of 0.9696. We obtained similar results from the swap experiment in which we swapped the training and validation sets and completely regenerated the factors and the predictive model (again maintaining strict independence of the switched training and validation sets), indicating the validity of the modeling approach based on the orthologs identified in mouse (Figure 3 D-F). The results of the mock factor analysis (Figure 3 G-I) confirm that a model based on a set of unrelated, biologically orthogonal factors is not sufficient to build a predictive model of breast tumor status. The mock factors, which were generated from an insulin/muscle-biopsy gene expression dataset (GSE 7146), were unable to distinguish between samples based on breast tumor status in the independent validation set. In addition to the swap experiment and mock factor analysis, we also performed permutation testing to evaluate the reproducibility of the predictive model. We used the same factors generated in the original training set, but randomly assigned samples to either the training or validation sets (100 permutations). The distribution of test characteristics from this analysis are shown in Additional File 4 and indicate that we are able to generate a robust predictive model regardless of which samples were assigned to the training and validation sets. Additional File 5 shows the results (overlaid with the results in Additional File 4 for comparison) of a negative control experiment in which we scrambled the phenotypic labels of the samples but used the same factors generated from the original training set to generate a potential predictive model. The distributions of test characteristics from these analyses show that the factors generated from the original training set are dependent on phenotype (presence of a tumor). Finally, to evaluate the possibility of generating a predictor derived purely from human peripheral blood mononuclear cell (PBMC) samples, we performed a sparse ANOVA (using identical parameters as those used with the mouse data) on a training cohort of patients with malignancy versus healthy volunteers with no malignant or benign breast lesions. This yielded a small subset of 81 genes. Employing BFRM as previously described to identify potentially predictive factors, we identified a single factor, which performed no better than the mouse-derived factors.

### Functional annotation of the genes comprising the breast tumor predictor

**Figure 4:**
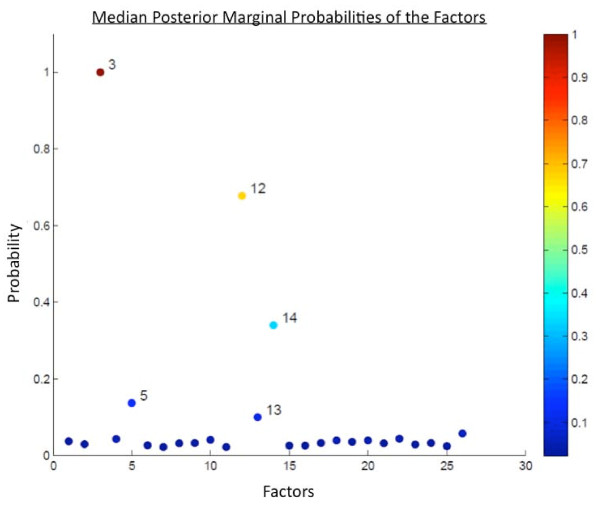
**Inclusion probabilities of factors in the predictive model**. We generated a collection of 26 factors from the human PBMC training set using the methods described previously and used SSS to put these together in various combinations to form predictive models (5000 iterations), which were validated in an independent sample set. We calculated the top performing factors based on their inclusion probability (median posterior marginal probability) in the top 200 models. These 26 factors are plotted along the x-axis and the median posterior marginal probabilities are plotted on the y-axis. The top 3 factors with inclusion probabilities significantly above the background noise are 3, 12 and 14.

The predictor described in this study reflects changes in the transcriptional program of the peripheral blood cells in response to the presence of a breast tumor. However, the mechanism(s) underlying this response are unknown and could be due to either a direct interaction between tumor and immune cells or indirect communication via signaling molecules. In order to better understand the biological underpinnings of this PBC response, we focused on the 3 top-performing factors based on their median posterior marginal probabilities, which were significantly above the level of background noise (Figure 4). These factors, designated Factor 3, Factor 12 and Factor 14, exhibit coordinated expression across the samples in the training set. In addition, the relationship of the genes in the factors is largely conserved in the validation set (Additional File 6). These factors are described in terms of their constituent Affymetrix probe identifiers in the Additional Data Files section (original_gene_factor_summary.txt). However, in order to simplify the presentation of these data, we have combined the gene lists of the three top factors in the predictor (3, 12 and 14) for a total of 115 unique probe identifiers (Additional File 7). Next, we analyzed this combined gene list based on the subsets of genes that were either upregulated (83 probe identifiers) or downregulated (32 probe identifiers) in the PBMCs of breast cancer patients compared to healthy controls using the various functions on GATHER (Gene Annotation Tool to Help Explain Relationships; [32]) and the GSEA (Gene Set Enrichment Analysis) Molecular Signatures Database [33,34]. The results of these analyses are included in the Additional Data Files section.

Using the GO (Gene Ontology) function of GATHER, we identified the following gene ontology categories that were significantly enriched in the upregulated subset of genes (p-value ≤ 0.003): I-kappaB kinase/nuclear factor (NF)-kappaB cascade (GO:0007249); protein kinase cascade (GO:0007243); intracellular signaling cascade (GO:0007242); positive regulation of I-kappaB kinase/NF-kappaB cascade (GO:0043123); regulation of I-kappaB kinase/NF-kappaB cascade (GO:0043122); pentose-phosphate shunt (GO:0006098); NADPH regeneration (GO:0006740); positive regulation of signal transduction (GO:0009967); NADPH metabolism (GO:0006739). We also identified similar gene ontology categories by calculating the overlap between the upregulated subset of genes within the predictor and GSEA gene ontology gene sets. Furthermore, the KEGG (Kyoto Encyclopedia of Genes and Genomes) analysis revealed that this subset of upregulated genes contained a significant number of genes involved in the canonical MAPK (mitogen-activated protein kinase) signaling pathway, which plays a role in NF-kappaB pathway signaling. In addition to the MAPK pathway, KEGG analysis also implicated the pentose phosphate pathway and the Jak-STAT (Janus kinase and signal transducer and activator of transcription) signaling pathway. The pentose phosphate pathway is involved in NADPH production and metabolism. This finding coincides with the initial GATHER GO analysis, which identified NADPH regeneration and metabolism categories as being significantly enriched within the upregulated genes in the predictor. The Jak-STAT signaling pathway is activated in response to interferon, interleukins and cytokines and results in the transcription of genes responsive to a particular STAT. In fact, the Protein Binding function of GATHER identified a significant enrichment of STAT3 binding partners within the upregulated set of genes and GSEA analysis reported significant overlap between the upregulated genes and gene sets related to IL-6 (interleukin 6), which is an activator of STAT3.

Using the TRANSFAC^® ^function of GATHER, we identified several transcription factor binding sites that were significantly enriched in the downregulated subset of genes (p-value ≤ 0.003). In particular, 3 of these transcription factors had putative binding sites in almost all of the downregulated genes in the predictor: 1) V$CETS1P54_01: c-Ets-1 (p54); 2) V$KROX_Q6; and 3) V$E2F_Q6: E2F. This subset of downregulated genes also contained 5 genes that overlapped with the peripheral blood signature of bladder cancer described by Osman et al. [3] (p-value = 0.000228).

We also compared the predictive models generated from the original experiment and the swap experiment based on factor annotation. The top factors in the predictive model from the swap experiment consisted of 457 unique Affymetrix probe sets (Additional File 8 and Additional File 9). Of these, 24 overlapped with the original model (115 probes). These 24 overlapping probe sets, or genes, are implicated in positive regulation of the I-kappaB kinase/NF-kappaB cascade, which strengthens the identification of this particular pathway in the original predictive model.

## Discussion

Previous work demonstrates the potential for measuring gene expression within peripheral blood as a means of assessing ongoing disease states. This includes the development of genomic analyses used to categorize and treat stroke [38,39], elucidation of mechanisms of autoimmune syndromes such as lupus [40] and rheumatoid arthritis [41] and, more recently, as a measure of viral infection, with the goal of early detection and better management of disease [42]. Several studies have extended the concept to cancer [1-7]. Our approach to developing a predictor of human breast cancer makes use of a transgenic mouse model system to first identify those genes most relevant to the presence of a tumor. As an experimental system, the mouse offers several advantages over human studies. It provides a homogeneous genetic background and the ability to control a number of variables, including environmental exposures and the presence of co-morbid conditions. Using this approach, we were able to identify a robust predictor that was capable of predicting tumor status in an independent validation set of mouse peripheral blood samples. We also used the transcriptome data generated through these mouse experiments as the starting point for building a predictor of human breast tumors. This predictor proved to be capable of distinguishing breast cancer patients from healthy individuals based on transcriptome patterns in the peripheral blood cells with a sensitivity of 89% and specificity of 100% in an independent validation cohort. Our results support the findings of Aarøe et al. [1] by identifying a pattern of gene expression that can distinguish breast cancer patients from healthy individuals with similar sensitivity and specificity. However, our predictor performs better in terms of sensitivity and specificity and does not overlap with their reported signature (with the exception of a single probe identifier, 221476_s_at, which corresponds to ribosomal protein L15).

Most importantly, our work represents a conceptual advancement in that we were able to link a mouse model of breast cancer with human breast cancer by applying a factor modeling approach to peripheral blood gene expression data. This suggests that the biological clarity of the mouse model system improves our ability to identify a common underlying biological response to the formation of the tumor. It also provides an experimental framework that can be used to pose new questions that cannot be easily addressed in human studies.

While the studies we report here do appear promising, we recognize that these represent first steps towards the goal of developing a blood-based predictor of human breast cancer. Further validation in a large, independent cohort is needed, as is validation in diverse populations. In addition, more research is needed to identify other potentially relevant factors that might not be represented in our particular study. We are also interested in exploring the specificity of this predictive model in terms of its ability to distinguish breast cancer from other types of tumors, as well as its specificity in distinguishing a breast tumor from other common conditions, such as infection. More importantly, we believe the approach described here offers a strategy to address important questions in the development of blood-based cancer diagnostics that are not easily addressed in human studies alone. The use of the mouse model system allows us to develop predictive models that often cannot be derived directly from heterogeneous clinical sample data and could potentially facilitate exploration of the presence of a tumor-associated complex biomarker in mice prior to evidence of a tumor. This approach could prove to be valuable in other contexts as well. A key example can be seen with ovarian cancer where the vast majority of patients are diagnosed with stage IV disease. Given that patients diagnosed with such advanced disease face a poor prognosis, the development of methods for detection at an early stage of the disease process would have a substantial impact on outcome.

## Conclusions

We have described a predictor of the presence of human breast tumors based on peripheral blood gene expression data. This predictor was generated using a well-defined transgenic mouse mammary tumor model as the starting point for identifying gene expression changes associated with the presence of a tumor. The predictor accurately distinguishes between samples based on the presence of breast tumors, which is significant within the field in two ways: first, the predictor demonstrates higher sensitivity and specificity than previously reported breast cancer signatures; second, it serves as a proof-of-concept for using mouse models of human cancer in the identification of potential clinical biomarkers. We believe that the approaches described here represent an experimental framework for better understanding the complexity of the peripheral blood cell response to solid tumors.

## Competing interests

J.R.N. has an ownership interest in Expression Analysis and has a consultant or advisory role in Qiagen.

## Authors' contributions

HGL designed and performed the murine and human experiments, interpreted the statistical analysis, developed functional biological annotations of the factors, and co-wrote the manuscript. JRN provided overall expertise in the design and interpretation of the experiments and analyses and provided critical analysis of the manuscript. EH designed and implemented QUADRA; performed and interpreted the statistical analysis; and co-wrote the manuscript. All authors have read and approved the final manuscript.

## Pre-publication history

The pre-publication history for this paper can be accessed here:

http://www.biomedcentral.com/1755-8794/4/61/prepub

## Supplementary Material

Additional File 1 **Expression levels of Myc transcript in peripheral blood**. The levels of Myc mRNA transcript in the peripheral blood were measured by quantitative RT-PCR in MMTV/c-myc mice of each of the five transgenic lines derived by Eran Andrechek. As a negative control, we analyzed expression levels in the peripheral blood and mammary gland of a wildtype lactating mouse. As a positive control, we analyzed expression levels in the tumor tissue from a transgenic mouse. Expression was normalized according to expression of the housekeeping gene beta-actin and set relative to the wildtype lactating mammary gland. (A) Total Myc levels were measured, including both endogenous and transgenic transcripts. (B) Levels of the Myc transgene alone were also measured, using primers specific to the transgenic construct. (C) Additionally, peripheral blood Myc transcript levels were calculated based on signal intensity of the Affymetrix probesets for the myelocytomatosis oncogene (1425923_at, 1417155_at,1425922_a_at, 1417155_at, 1425922_a_at, 1425923_at, and 1424942_a_at). There were no significant differences in expression levels for any of the three probes across all three classes of mouse. Light blue = wildtype FVB virgin mice; dark blue = wildtype FVB age-matched controls; pink = MMTV/c-myc transgenic mice prior to tumor palpation; red = MMTV/c-myc transgenic tumor-bearing mice. Click here for file

Additional File 2 **Analysis of leukocyte subpopulations in mouse peripheral blood**. Samples were collected from mice by venipuncture from the hepatic portal vein following euthanization in BD Microtainer™ tubes with potassium-EDTA anticoagulant, placed on ice and analyzed within 8 hours. The Duke University Medical Center Veterinary Diagnostic Laboratory analyzed samples using a CELL-DYN 3700 Hematology Analyzer. Leukocyte subpopulations were counted and calculated as a percentage of total leukocytes in each cohort of mice: virgin control mice (n = 4); controls matched for age and parity (n = 15); transgenic mice with advanced tumors (n = 28); and wildtype mice with MMTV/c-myc tumor implants that have reached approximately 1 cm in diameter (n = 5). The Virgin Control mice, which were considered to be immunologically naïve, have a distinctly different distribution of leukocytes subgroups. However, all other groups of mice show similar leukocyte profiles, indicating that any gene expression differences observed are likely a result of the presence of the tumor, rather than differences in the proportion of a particular cell type. Click here for file

Additional File 3 **Characteristics of Normal vs. Malignant samples**. Table comparing the demographic and clinical variables of the Normal and Malignant samples. Click here for file

Additional File 4 **Distribution of test characteristics from data set permutation testing**. We used the factors generated from the original training set, but randomly assigned samples to either the training or validation set (100 permutations) and plotted the distribution of the following test characteristics: p-value (A), sensitivity (B), specificity (C) and AUC (D). Click here for file

Additional File 5 **Distribution of test characteristics from phenotype permutation testing**. We used the factors generated from the original training set, but phenotypic labels of the samples were randomly permuted (200 iterations) and plotted the distribution of the following test characteristics: p-value (A), sensitivity (B), specificity (C) and AUC (D). Black = original phenotypes and red = scrambled phenotypes. Click here for file

Additional File 6 **Factor coherence between the training and validation sets**. Each of the top 3 factors that compose the human breast cancer predictor (3, 12 and 14) exhibit coordinated gene expression across the training set (A, C and E). Furthermore, this coordinate expression is recapitulated in the validation set (B, D and F). Each column represents a human PBMC sample. Samples are ordered left to right in descending order of their loading on the 1st principal component. Each row is a gene (probe set) in descending order of correlation. Red = high expression and yellow = low expression. Click here for file

Additional File 7 **Inclusion probabilities of factors in the swapped model**. We generated a collection of 50 factors from the human PBMC training set using the methods described previously and used SSS to put these together in various combinations to form predictive models (5000 iterations), which were validated in an independent sample set. We calculated the top performing factors based on their inclusion probability (posterior marginal probability) in the top 200 models. These 50 factors are plotted along the x-axis and the median posterior marginal probabilities are plotted on the y-axis. The top 5 factors with inclusion probabilities significantly above the background noise are 13, 25, 26, 20 and 42. Click here for file

Additional File 8 **Constituent probe identifiers of the top 3 factors of the breast cancer predictor**. Table containing the Affymetrix probe identifiers of each of the top 3 factors identified in the study. Click here for file

Additional File 9 **BMC_Miniwebsite **Tabular documents generated from the functional annotation of the top 3 factors. Click here for file

## References

[B1] Aaroe J Lindahl T Dumeaux V Saebo S Tobin D Hagen N Skaane P Lonneborg A Sharma P Borresen-Dale AL Gene expression profiling of peripheral blood cells for early detection of breast cancer Breast Cancer Res 2010 12 R7 10.1186/bcr2472 20078854 PMC2880427

[B2] Han M Liew CT Zhang HW Chao S Zheng R Yip KT Song ZY Li HM Geng XP Zhu LX Novel blood-based, five-gene biomarker set for the detection of colorectal cancer Clin Cancer Res 2008 14 455 460 10.1158/1078-0432.CCR-07-1801 18203981

[B3] Osman I Bajorin DF Sun TT Zhong H Douglas D Scattergood J Zheng R Han M Marshall KW Liew CC Novel blood biomarkers of human urinary bladder cancer Clin Cancer Res 2006 12 3374 3380 10.1158/1078-0432.CCR-05-2081 16740760

[B4] Sharma P Sahni NS Tibshirani R Skaane P Urdal P Berghagen H Jensen M Kristiansen L Moen C Zaka A Early detection of breast cancer based on gene-expression patterns in peripheral blood cells Breast Cancer Res 2005 7 R634 644 10.1186/bcr1203 16168108 PMC1242124

[B5] Showe MK Vachani A Kossenkov AV Yousef M Nichols C Nikonova EV Chang C Kucharczuk J Tran B Wakeam E Gene expression profiles in peripheral blood mononuclear cells can distinguish patients with non-small cell lung cancer from patients with nonmalignant lung disease Cancer Res 2009 69 9202 9210 10.1158/0008-5472.CAN-09-1378 19951989 PMC2798582

[B6] Twine NC Stover JA Marshall B Dukart G Hidalgo M Stadler W Logan T Dutcher J Hudes G Dorner AJ Disease-associated expression profiles in peripheral blood mononuclear cells from patients with advanced renal cell carcinoma Cancer Res 2003 63 6069 6075 14522937

[B7] Xu T Shu CT Purdom E Dang D Ilsley D Guo Y Weber J Holmes SP Lee PP Microarray analysis reveals differences in gene expression of circulating CD8(+) T cells in melanoma patients and healthy donors Cancer Res 2004 64 3661 3667 10.1158/0008-5472.CAN-03-3396 15150126

[B8] Bild AH Yao G Chang JT Wang Q Potti A Chasse D Joshi MB Harpole D Lancaster JM Berchuck A Oncogenic pathway signatures in human cancers as a guide to targeted therapies Nature 2006 439 353 357 10.1038/nature04296 16273092

[B9] Huang E Cheng SH Dressman H Pittman J Tsou MH Horng CF Bild A Iversen ES Liao M Chen CM Gene expression predictors of breast cancer outcomes Lancet 2003 361 1590 1596 10.1016/S0140-6736(03)13308-9 12747878

[B10] Huang E Ishida S Pittman J Dressman H Bild A Kloos M D'Amico M Pestell RG West M Nevins JR Gene expression phenotypic models that predict the activity of oncogenic pathways Nat Genet 2003 34 226 230 10.1038/ng1167 12754511

[B11] Kamangar F Dores GM Anderson WF Patterns of cancer incidence, mortality, and prevalence across five continents: defining priorities to reduce cancer disparities in different geographic regions of the world J Clin Oncol 2006 24 2137 2150 10.1200/JCO.2005.05.2308 16682732

[B12] SEER Cancer Statistics Review, 1975-2007 http://seer.cancer.gov/csr/1975_2008/index.html

[B13] Weedon-Fekjaer H Lindqvist BH Vatten LJ Aalen OO Tretli S Breast cancer tumor growth estimated through mammography screening data Breast Cancer Res 2008 10 R41 10.1186/bcr2092 18466608 PMC2481488

[B14] Porter PL El-Bastawissi AY Mandelson MT Lin MG Khalid N Watney EA Cousens L White D Taplin S White E Breast tumor characteristics as predictors of mammographic detection: comparison of interval- and screen-detected cancers J Natl Cancer Inst 1999 91 2020 2028 10.1093/jnci/91.23.2020 10580027

[B15] Elmore JG Armstrong K Lehman CD Fletcher SW Screening for breast cancer JAMA 2005 293 1245 1256 10.1001/jama.293.10.1245 15755947 PMC3149836

[B16] Kerlikowske K Grady D Barclay J Sickles EA Ernster V Likelihood ratios for modern screening mammography. Risk of breast cancer based on age and mammographic interpretation JAMA 1996 276 39 43 10.1001/jama.276.1.39 8667537

[B17] Kolb TM Lichy J Newhouse JH Comparison of the performance of screening mammography, physical examination, and breast US and evaluation of factors that influence them: an analysis of 27,825 patient evaluations Radiology 2002 225 165 175 10.1148/radiol.2251011667 12355001

[B18] Rosenberg RD Hunt WC Williamson MR Gilliland FD Wiest PW Kelsey CA Key CR Linver MN Effects of age, breast density, ethnicity, and estrogen replacement therapy on screening mammographic sensitivity and cancer stage at diagnosis: review of 183,134 screening mammograms in Albuquerque, New Mexico Radiology 1998 209 511 518 9807581 10.1148/radiology.209.2.9807581

[B19] Amir E Freedman OC Seruga B Evans DG Assessing women at high risk of breast cancer: a review of risk assessment models J Natl Cancer Inst 2010 102 680 691 10.1093/jnci/djq088 20427433

[B20] Warner E Plewes DB Hill KA Causer PA Zubovits JT Jong RA Cutrara MR DeBoer G Yaffe MJ Messner SJ Surveillance of BRCA1 and BRCA2 mutation carriers with magnetic resonance imaging, ultrasound, mammography, and clinical breast examination JAMA 2004 292 1317 1325 10.1001/jama.292.11.1317 15367553

[B21] Andrechek ER Cardiff RD Chang JT Gatza ML Acharya CR Potti A Nevins JR Genetic heterogeneity of Myc-induced mammary tumors reflecting diverse phenotypes including metastatic potential Proc Natl Acad Sci USA 2009 106 16387 16392 10.1073/pnas.0901250106 19805309 PMC2752567

[B22] Leder A Pattengale PK Kuo A Stewart TA Leder P Consequences of widespread deregulation of the c-myc gene in transgenic mice: multiple neoplasms and normal development Cell 1986 45 485 495 10.1016/0092-8674(86)90280-1 3011271

[B23] Affymetrix NetAffx http://www.affymetrix.com/analysis/index.affx

[B24] Lucas J Carvalho C West M A bayesian analysis strategy for cross-study translation of gene expression biomarkers Stat Appl Genet Mol Biol 2009 8 Article 11 19222378 10.2202/1544-6115.1436 PMC2861325

[B25] Carvalho CM Lucas JE Wang Q Chang J Nevins JR West M High-dimensional sparse factor modelling - Applicaitons in gene expression genomics Journal of the American Statistical Association 2008 103 1438 1456 10.1198/016214508000000869 21218139 PMC3017385

[B26] Hans C Shotgun stochastic search for "Large p" regression Journal of the American Statistical Association 2007 102 507 516 10.1198/016214507000000121

[B27] Hoeting JA Bayesian Model Averaging: A Tutorial Statistical Science 1999 14 382 417 10.1214/ss/1009212519

[B28] QUADRA http://quadra.genome.duke.edu/mouse-2-human-mammary-tumor-pbmc-project

[B29] NCBI Gene Expression Omnibus http://www.ncbi.nlm.nih.gov/geo/query/acc.cgi?acc = GSE27567

[B30] Shi L Campbell G Jones WD Campagne F Wen Z Walker SJ Su Z Chu TM Goodsaid FM Pusztai L The MicroArray Quality Control (MAQC)-II study of common practices for the development and validation of microarray-based predictive models Nat Biotechnol 2010 28 827 838 10.1038/nbt.1665 20676074 PMC3315840

[B31] GATHER http://gather.genome.duke.edu/

[B32] Chang JT Nevins JR GATHER: a systems approach to interpreting genomic signatures Bioinformatics 2006 22 2926 2933 10.1093/bioinformatics/btl483 17000751

[B33] Subramanian A Kuehn H Gould J Tamayo P Mesirov JP GSEA-P: a desktop application for Gene Set Enrichment Analysis Bioinformatics 2007 23 3251 3253 10.1093/bioinformatics/btm369 17644558

[B34] Subramanian A Tamayo P Mootha VK Mukherjee S Ebert BL Gillette MA Paulovich A Pomeroy SL Golub TR Lander ES Mesirov JP Gene set enrichment analysis: a knowledge-based approach for interpreting genome-wide expression profiles Proc Natl Acad Sci USA 2005 102 15545 15550 10.1073/pnas.0506580102 16199517 PMC1239896

[B35] Gene Set Enrichment Analysis http://www.broadinstitute.org/gsea/msigdb/annotate.jsp

[B36] Git http://www.git-scm.org

[B37] Gitorious http://www.gitorious.com

[B38] Baird AE Blood genomics in human stroke Stroke 2007 38 694 698 10.1161/01.STR.0000250431.99687.7b 17261718

[B39] Xu H Gene expression in peripheral blood differs after cardioembolic compared with large-vessel atherosclerotic stroke: biomarkers for the etiology of ischemic stroke Journal of Cerebral Blood Flow and Metabolism 2008 28 1320 1328 10.1038/jcbfm.2008.22 18382470

[B40] Bennett L Palucka AK Arce E Cantrell V Borvak J Banchereau J Pascual V Interferon and granulopoiesis signatures in systemic lupus erythematosus blood J Exp Med 2003 197 711 723 10.1084/jem.20021553 12642603 PMC2193846

[B41] Batliwalla FM Baechler EC Xiao X Li W Balasubramanian S Khalili H Damle A Ortmann WA Perrone A Kantor AB Peripheral blood gene expression profiling in rheumatoid arthritis Genes Immun 2005 6 388 397 10.1038/sj.gene.6364209 15973463

[B42] Zaas AK Chen M Varkey J Veldman T Hero AO Lucas J Huang Y Turner R Gilbert A Lambkin-Williams R Gene expression signatures diagnose influenza and other symptomatic respiratory viral infections in humans Cell Host Microbe 2009 6 207 217 10.1016/j.chom.2009.07.006 19664979 PMC2852511

