# Supplementary figures and images for: Integrating Factor Analysis and a Transgenic Mouse Model to Reveal a Peripheral Blood Predictor of Breast Tumors
Source: BMC Med Genomics. 2011 Jul 22;4:61. doi: 10.1186/1755-8794-4-61 (PMC3178481; doi:10.1186/1755-8794-4-61)

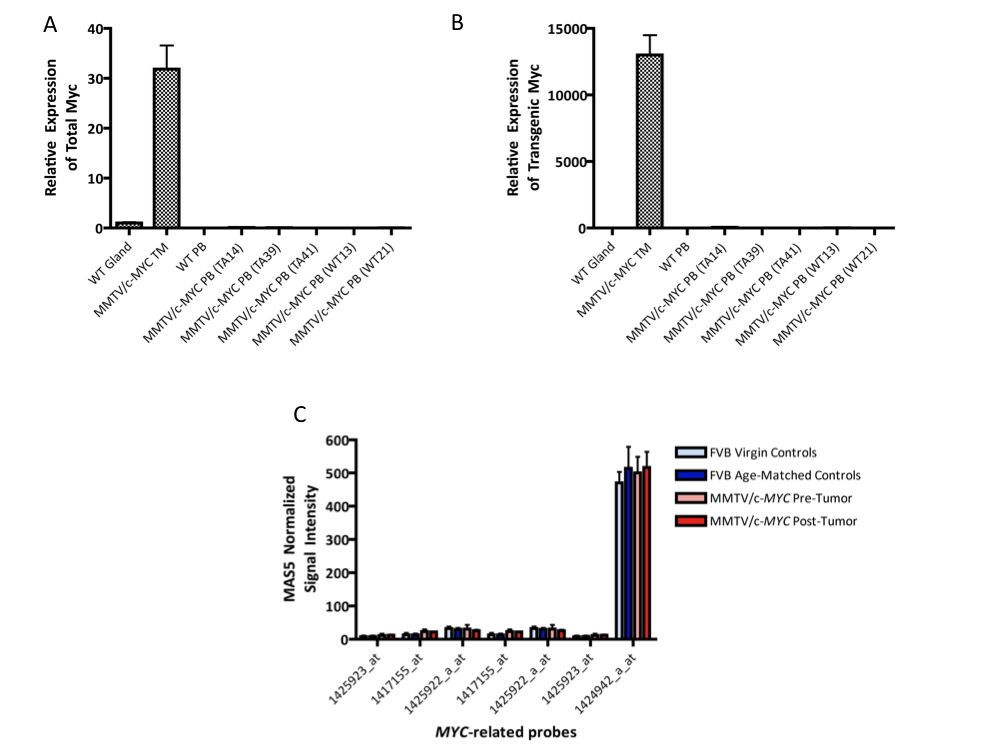

Supplement: Additional File 1 — Expression levels of Myc transcript in peripheral blood. The levels of Myc mRNA transcript in the peripheral blood were measured by quantitative RT-PCR in MMTV/c-myc mice of each of the five transgenic lines derived by Eran Andrechek. As a negative control, we analyzed expression levels in the peripheral blood and mammary gland of a wildtype lactating mouse. As a positive control, we analyzed expression levels in the tumor tissue from a transgenic mouse. Expression was normalized according to expression of the housekeeping gene beta-actin and set relative to the wildtype lactating mammary gland. (A) Total Myc levels were measured, including both endogenous and transgenic transcripts. (B) Levels of the Myc transgene alone were also measured, using primers specific to the transgenic construct. (C) Additionally, peripheral blood Myc transcript levels were calculated based on signal intensity of the Affymetrix probesets for the myelocytomatosis oncogene (1425923_at, 1417155_at,1425922_a_at, 1417155_at, 1425922_a_at, 1425923_at, and 1424942_a_at). There were no significant differences in expression levels for any of the three probes across all three classes of mouse. Light blue = wildtype FVB virgin mice; dark blue = wildtype FVB age-matched controls; pink = MMTV/c-myc transgenic mice prior to tumor palpation; red = MMTV/c-myc transgenic tumor-bearing mice. [file 1755-8794-4-61-S1.TIFF]

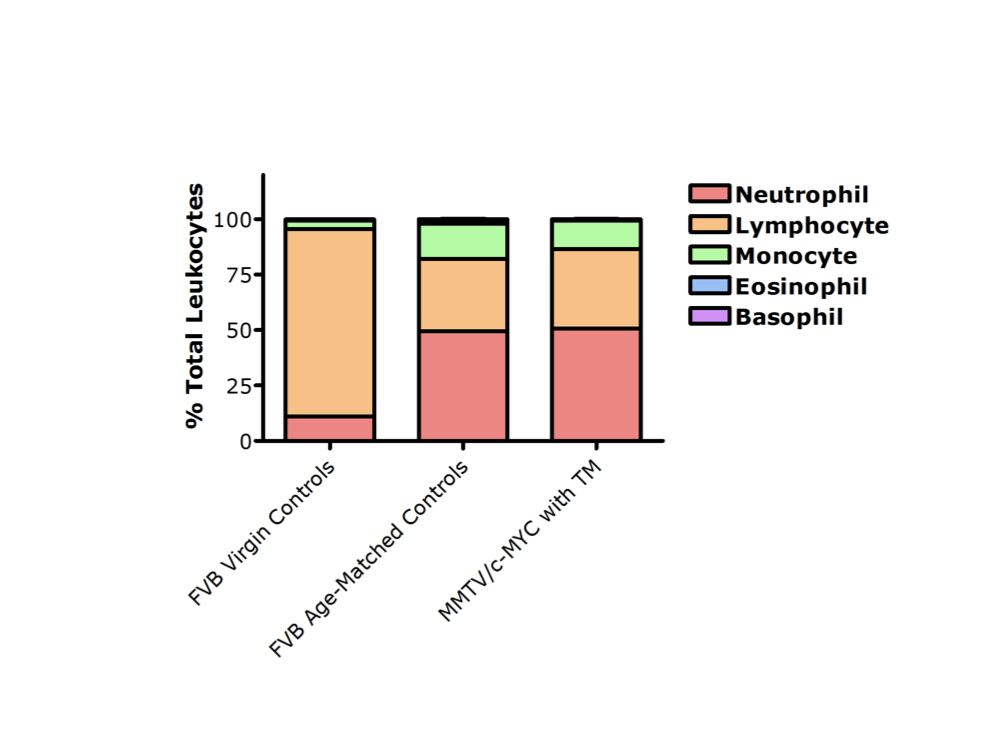

Supplement: Additional File 2 — Analysis of leukocyte subpopulations in mouse peripheral blood. Samples were collected from mice by venipuncture from the hepatic portal vein following euthanization in BD Microtainer™ tubes with potassium-EDTA anticoagulant, placed on ice and analyzed within 8 hours. The Duke University Medical Center Veterinary Diagnostic Laboratory analyzed samples using a CELL-DYN 3700 Hematology Analyzer. Leukocyte subpopulations were counted and calculated as a percentage of total leukocytes in each cohort of mice: virgin control mice (n = 4); controls matched for age and parity (n = 15); transgenic mice with advanced tumors (n = 28); and wildtype mice with MMTV/c-myc tumor implants that have reached approximately 1 cm in diameter (n = 5). The Virgin Control mice, which were considered to be immunologically naïve, have a distinctly different distribution of leukocytes subgroups. However, all other groups of mice show similar leukocyte profiles, indicating that any gene expression differences observed are likely a result of the presence of the tumor, rather than differences in the proportion of a particular cell type. [file 1755-8794-4-61-S2.TIFF]

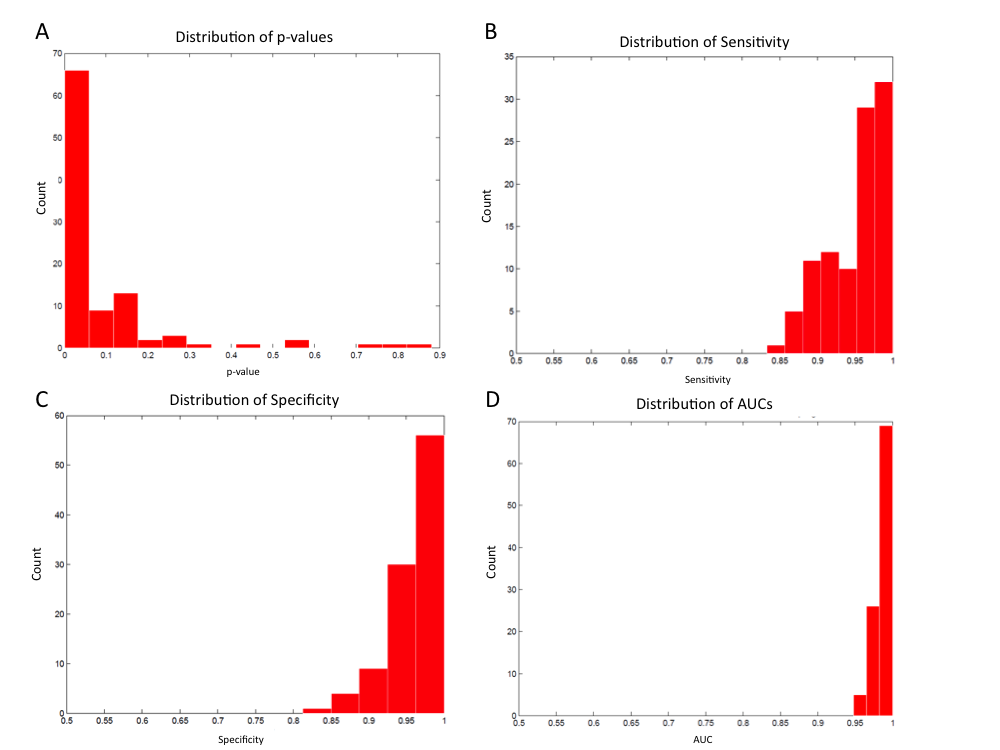

Supplement: Additional File 3 — Characteristics of Normal vs. Malignant samples. Table comparing the demographic and clinical variables of the Normal and Malignant samples. [file 1755-8794-4-61-S3.TIFF]

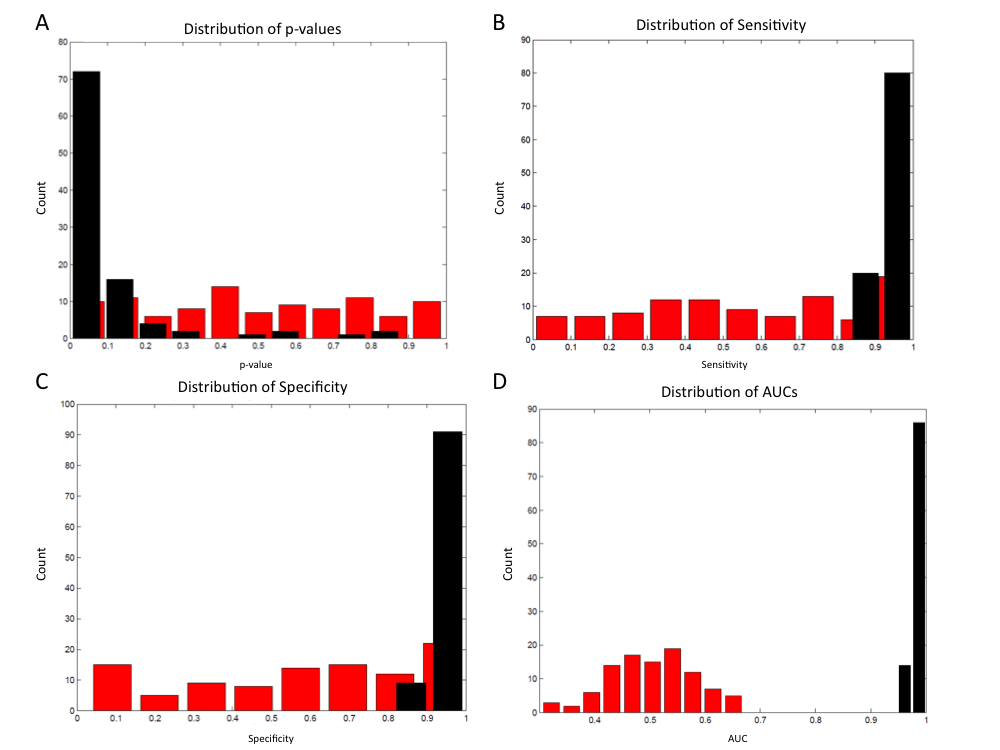

Supplement: Additional File 4 — Distribution of test characteristics from data set permutation testing. We used the factors generated from the original training set, but randomly assigned samples to either the training or validation set (100 permutations) and plotted the distribution of the following test characteristics: p-value (A), sensitivity (B), specificity (C) and AUC (D). [file 1755-8794-4-61-S4.TIFF]

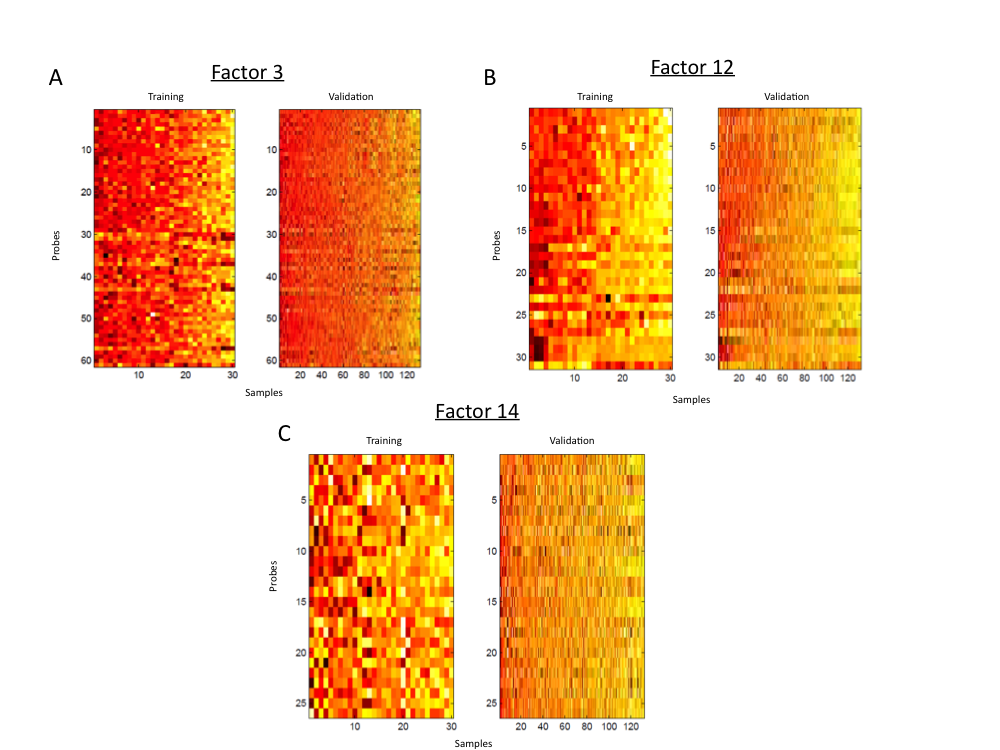

Supplement: Additional File 5 — Distribution of test characteristics from phenotype permutation testing. We used the factors generated from the original training set, but phenotypic labels of the samples were randomly permuted (200 iterations) and plotted the distribution of the following test characteristics: p-value (A), sensitivity (B), specificity (C) and AUC (D). Black = original phenotypes and red = scrambled phenotypes. [file 1755-8794-4-61-S5.TIFF]

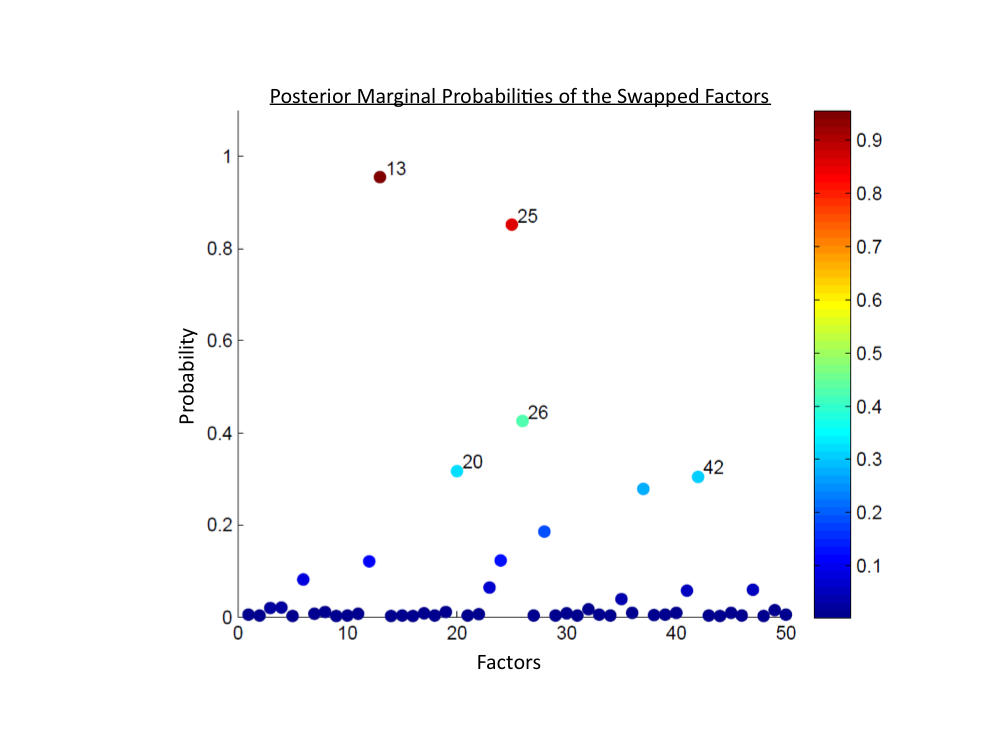

Supplement: Additional File 6 — Factor coherence between the training and validation sets. Each of the top 3 factors that compose the human breast cancer predictor (3, 12 and 14) exhibit coordinated gene expression across the training set (A, C and E). Furthermore, this coordinate expression is recapitulated in the validation set (B, D and F). Each column represents a human PBMC sample. Samples are ordered left to right in descending order of their loading on the 1st principal component. Each row is a gene (probe set) in descending order of correlation. Red = high expression and yellow = low expression. [file 1755-8794-4-61-S6.TIFF]

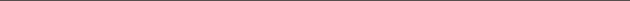

Supplement: Additional File 9 — BMC_Miniwebsite Tabular documents generated from the functional annotation of the top 3 factors. [file 1755-8794-4-61-S9.ZIP › BMC_MiniWebsite/Index_files/shapeimage_1.jpg]

Supplement: Additional File 9 — BMC_Miniwebsite Tabular documents generated from the functional annotation of the top 3 factors. [file 1755-8794-4-61-S9.ZIP › BMC_MiniWebsite/Media/transparent.gif]

Supplement: Additional File 9 — BMC_Miniwebsite Tabular documents generated from the functional annotation of the top 3 factors. [file 1755-8794-4-61-S9.ZIP › BMC_MiniWebsite/Scripts/Widgets/SharedResources/None.gif]

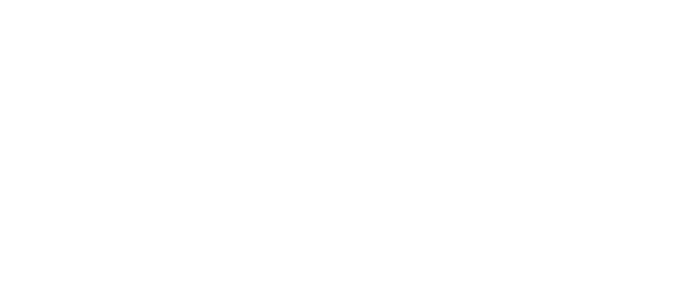

Supplement: Additional File 9 — BMC_Miniwebsite Tabular documents generated from the functional annotation of the top 3 factors. [file 1755-8794-4-61-S9.ZIP › BMC_MiniWebsite/Scripts/Widgets/SharedResources/Translucent-Overlay.png]
