# Supplementary material for: Integrating Factor Analysis and a Transgenic Mouse Model to Reveal a Peripheral Blood Predictor of Breast Tumors
Source: BMC Med Genomics. 2011 Jul 22;4:61. doi: 10.1186/1755-8794-4-61 (PMC3178481; doi:10.1186/1755-8794-4-61)
Supplement: Additional File 9 — BMC_Miniwebsite Tabular documents generated from the functional annotation of the top 3 factors. [file 1755-8794-4-61-S9.ZIP › BMC_MiniWebsite/Index.html]

xml version="1.0" encoding="UTF-8"?


MINIWEBSITE for
“Integrating Factor Analysis and a Transgenic Mouse Model to Reveal a Peripheral Blood Predictor of Breast Tumors”


MINIWEBSITE for

“Integrating Factor Analysis and a Transgenic Mouse Model to Reveal a Peripheral Blood Predictor of Breast Tumors”

Duke University, Durham, NC

Sage Bionetworks, Seattle, WA

Supplemental files for this manuscript are maintained here. Please see the navigation bar above for links to downloadable supplementary files.

When downloading files, please right-click and “Save as...” to your local machine.

If you don’t see the menu bar above, use these links:

Sparse ANOVA

Original Factor Model

Swapped Factor Model

AUTHORS: Heather G. LaBreche, Joseph R. Nevins, Erich S. Huang
