# Supplementary material for: Integrating Factor Analysis and a Transgenic Mouse Model to Reveal a Peripheral Blood Predictor of Breast Tumors
Source: BMC Med Genomics. 2011 Jul 22;4:61. doi: 10.1186/1755-8794-4-61 (PMC3178481; doi:10.1186/1755-8794-4-61)
Supplement: Additional File 9 — BMC_Miniwebsite Tabular documents generated from the functional annotation of the top 3 factors. [file 1755-8794-4-61-S9.ZIP › BMC_MiniWebsite/Original_Factor_Model.html]

xml version="1.0" encoding="UTF-8"?


Original Factor Model


Original Factor Model

DN\_Top3\_MPM\_gather\_transfac.txt

DN\_Top3\_MPMP\_gather\_gene\_ontology.txt

DN\_Top3\_MPMP\_gather\_kegg.txt

DN\_Top3\_MPMP\_gather\_maploc.txt

DN\_Top3\_MPMP\_gather\_proteins.txt

DN\_Top3\_MPMP\_GSEA\_C1.txt

DN\_Top3\_MPMP\_GSEA\_C2.txt

DN\_Top3\_MPMP\_GSEA\_C3.txt

DN\_Top3\_MPMP\_GSEA\_C4.txt

DN\_Top3\_MPMP\_GSEA\_C5.txt

DN\_Top3Factors.txt

gene\_factor\_summary.txt

original\_gene\_factor\_summary.txt

UP\_Top3\_MPMP\_C1.txt

UP\_Top3\_MPMP\_C2.txt

UP\_Top3\_MPMP\_C3.txt

UP\_Top3\_MPMP\_C4.txt

UP\_Top3\_MPMP\_C5.txt

UP\_Top3\_MPMP\_gather\_gene\_ontology.txt

UP\_Top3\_MPMP\_gather\_kegg.txt

UP\_Top3\_MPMP\_gather\_maploc.txt

UP\_Top3\_MPMP\_gather\_proteins.txt

UP\_Top3\_MPMP\_gather\_transfac.txt

UP\_Top3Factors.txt

If you don’t see the menu bar above, use these links:

Index

Sparse ANOVA

Original Factor Model

Swapped Factor Model
