# Supplementary material for: Integrating Factor Analysis and a Transgenic Mouse Model to Reveal a Peripheral Blood Predictor of Breast Tumors
Source: BMC Med Genomics. 2011 Jul 22;4:61. doi: 10.1186/1755-8794-4-61 (PMC3178481; doi:10.1186/1755-8794-4-61)
Supplement: Additional File 9 — BMC_Miniwebsite Tabular documents generated from the functional annotation of the top 3 factors. [file 1755-8794-4-61-S9.ZIP › BMC_MiniWebsite/Swapped_Factor_Model.html]

xml version="1.0" encoding="UTF-8"?


Swapped Factor Model


Swapped Factor Model

gene\_factor\_summary.txt

swapped\_gene\_factor\_summary.txt

If you don’t see the menu bar above, use these links:

Index

Sparse ANOVA

Original Factor Model

Swapped Factor Model
